# Development prospects of “Internet + medical and health services” in Shandong: application status and public acceptance

**DOI:** 10.3389/fpubh.2025.1589224

**Published:** 2025-08-06

**Authors:** YingJie Gao, YaQiong Wang, XiaoLin Yan, GuanNan Sun, Cong Chen, Xiang Gao

**Affiliations:** ^1^Maternity & Child Care Center of DeZhou (DeZhou Women's & Children's Hospita), Dezhou, Shangdong, China; ^2^Dezhou Health Development Center, Dezhou, Shangdong, China

**Keywords:** survey, questionnaire, application status, acceptance, Internet

## Abstract

**Background:**

The integration of Internet technology into healthcare has led to the birth of the “Internet + medical and health services” model-a transformative approach to improving accessibility and efficiency in healthcare delivery.

**Objective:**

This study aims to assess the current status, challenges, and user perceptions of the “Internet + medical and health” services platform among individuals in Shandong, China.

**Methods:**

The cross-sectional survey was conducted between September 2023 and October 2023 on patients from public medical institutions in Shandong Province. A structured questionnaire was employed to gather information on the general characteristics of the respondents and information about platform usage and user experiences.

**Results:**

Of the 400 distributed questionnaires, 353 were valid. Most respondents were female (59.38%), aged 31–35 (28.13%), and held a bachelor's degree (55.97%). Compared to the educational distribution in the census results, individuals with a bachelor's degree or equivalent were significantly overrepresented in the surveyed population (16% vs. 69.61%, *p* < 0.001). Key barriers included a preference for face-to-face consultations (18%) and data security concerns (15%). Respondents emphasized the need for clearer legal regulations (4.33 ± 0.73) and greater public awareness (4.23 ± 0.68).

**Conclusions:**

People with higher education levels are more likely to adopt Internet Plus healthcare services, noting the hospital-affiliated platforms as their most-used types. While users appreciate the benefits of digital healthcare, concerns regarding personal information security and regulatory clarity continue to obstruct their wider use. Boosting data security, developing legal frameworks, and increasing user education should be the three central areas of improvement to facilitate a greater degree of acceptance and successful implementation of Internet Plus healthcare services.

## Introduction

With the popularization of Internet technology, the enhancement of bandwidth/speed, and the advent of new tools such as mobile payment, remote video, and the Internet of Things, Internet-based medical care has also seen rapid development (1, 2). The term “Internet + medical and health services” refers to the reliance on the Internet platform and the use of technologies such as the Internet of Things, cloud computing, big data, and mobile communications to facilitate “remote” consultation, online drug purchase, online medical insurance settlement, and other health services (3–5). This is a combination of Internet technology and an innovative product that is deeply integrated with traditional medical and health services.

Medical and health services are intrinsically related to the health and wellbeing of the people. With the rising awareness of national health care, residents have increasingly higher expectations for medical and health services in the new era (6–8). Currently, the “Internet + medical and health services” model has become a new driving force and a fresh direction to foster the development of medical and health services. This innovative technology can effectively reduce geographical barriers and enhance access to medical diagnosis and treatment in resource-limited areas (9–11). It can also improve the utilization efficiency of medical resources. For medical institutions, it is imperative that major hospitals continuously improve their medical informatization level and adhere to the goals of enhancing patient medical experience, boosting work efficiency, eliminating medical errors, and reducing operating costs (12, 13).

In late 2019 and early 2020, in response to the COVID-19 pandemic, owing to conflicts between restrictions on social activities and general medical diagnosis and treatment, the scope, quantity, and frequency of the utilization of Internet + medical health services have seen a rapid global increase (14–16). Researchers now believe that digital health services can play a significant role beyond the COVID-19 pandemic period, potentially helping to narrow the disparity in medical quality between regions, facilitate the decentralization of medical resources, and reduce patients' time and costs for healthcare. Thus far, healthcare seekers, particularly the younger demographic, have largely accepted and adapted to the digital health service model. These services include online registration, medical record access and duplication, remote medical consultation or re-examination, home delivery of medications, and contracted family doctor consultations, among others (17, 18). However, information is still lacking on the current status of service provision and user engagement on digital health platforms, as well as the challenges faced by patients in the post-COVID-19 era.

Existing studies focus on the technical and operational aspects of “Internet + medical and health services” (19), but limited research explores user adoption, service effectiveness, and public perceptions—especially within specific regional contexts like Shandong, China. Furthermore, barriers such as data security concerns, regulatory clarity, and user reluctance remain insufficiently addressed. This study seeks to fill these gaps by systematically examining the current adoption status, challenges, and user perceptions of the “Internet + medical and health services” platform in Shandong, China. The study will explore factors influencing adoption, common usage patterns, barriers to engagement, and key areas for improvement. The findings will contribute to the optimization of digital healthcare services, offering recommendations for enhancing public trust, strengthening data security, and refining regulatory frameworks to support wider adoption. By systematically analyzing these aspects, this research seeks to bridge the knowledge gap in digital healthcare adoption and provide actionable insights for policymakers, healthcare institutions, and technology developers.

## Methods

### Participants

The research subjects are patients in public medical institutions in Shandong Province from September 2023 to October 2023. A survey was conducted on the application status and recognition of user-friendly “Internet + medical and health services” platforms. The survey primarily employed interviews and online questionnaires filled out by investigators (https://www.wjx.cn/xz/239891564.aspx). Eighty patients were interviewed at each of the five hospitals. The questionnaires were collected, and those with a too-short response time (< 1 min) or incomplete were eliminated. A total of 352 valid questionnaires were obtained, yielding a response rate of 88%.

### Selection of the participants

The inclusion criteria for patient selection were as follows: (1) outpatients aged 14 years or older who visited the hospital and were able to clearly articulate their personal views; (2) patients who voluntarily participated and provided informed consent. The exclusion criteria included patients who explicitly declined participation.

### Questionnaire design

Before conducting the survey, we established a dedicated survey team. The team members underwent comprehensive training conducted by the researchers, covering the survey background, questionnaire design, and key considerations. During the questionnaire development process, we structured it into two distinct sections. The first section collected basic demographic details of the respondents. The second section consisted of a scenario-based simulation where respondents selected from multiple sets of options within predefined scenarios. The options were primarily derived from published studies on Internet-based healthcare and patient satisfaction (20–22). The questionnaire was finalized following this design process.

### Questionnaire construction and contents

Our questionnaire has four sections, including questions about demographic information, family relatedness, the type of platform being used, and the person understands of the platform. The demographic section obtained information on the gender, age, occupation, existing type of medical insurance, permanent residence, and monthly income status of participants. In addition, some data useful in determining the family circumstances of participants, such as the number of children, the age of the eldest child, and that of the oldest parent, were collected. The survey also inquires about the type of platform that participants are using and which “Internet + medical health” service platforms. A multiple-choice question asks, “From the following choices, which reason described would best account for the participants not having used or continuing to use the Internet + medical health service?” Recognition of the platform this survey would consist of 17 items about possible convenience advantages of “Internet + medical and health” over traditional medical means, with each item rated on a 5-point Likert scale from “completely agree” (5 points) to “completely disagree” (1 point). The survey methodology utilized best practices in question construction to ensure neutrality and prevent response bias. Before finalization, a pilot test was conducted with 30 participants, and based on their feedback, some ambiguous wording was refined. Particularly, in preparing the multiple-choice question on reasons for non-usage of Internet + healthcare services, balanced responses were included so that, typically, no particular choice would lead to a preferred answer. The Cronbach's alpha coefficient is 0.912, indicating high reliability.

### Sample size calculation

This study follows the standard method for calculating the required sample size for a questionnaire survey when the population proportion is known.


$$\begin{array}{l} n=\frac{Z^{2}\cdot p\left(1-p\right)}{E^{2}} \end{array}$$

The calculation formula is presented above, with the overall proportion based on China's 2023 Internet penetration rate. According to the Statistical Report on Internet Development in China issued by the China Internet Network Information Center, the Internet penetration rate in China was 77.5% as of December 2023 (https://www.cnnic.net.cn/n4/2024/0322/c88-10964.html). The key parameters used in this calculation are as follows: the standard normal deviation (*Z*) = 1.96, the estimated proportion (*p*) = 0.775, and the allowable margin of error (*E*) = 0.05. Based on these values, the required sample size is ~278 cases. However, to account for a questionnaire response rate of 80% and an expected dropout rate of 10%, the final planned sample size is 387. Participants were randomly selected from five medical centers, with a target of ~80 respondents per center.

### Statistical analysis

Statistical analysis was conducted using SPSS version 25.0 (IBM Corporation, NY, USA). To evaluate reliability of questionnaire, Cronbach's alpha coefficient was calculated. The Shapiro-Wilk normality test was used to assess whether the data followed a normal distribution. Variables that did not meet the normality assumption were reported as the median and interquartile range (Q1–Q3), while differences between two groups were analyzed using the Wilcoxon rank-sum test. For numerical variables that followed a normal distribution, data were presented as mean ± standard deviation (SD), and group differences were assessed using an independent sample *t*-test. Categorical variables were expressed as frequencies and proportions, with differences between groups analyzed using the chi-square test. A *p*-value of < 0.05 was considered statistically significant.

## Results

### Basic characteristic

A total of 400 questionnaires were collected, out of which 353 were deemed suitable for analysis. An analysis of the socio-economic attributes of the survey group revealed a higher number of female respondents, constituting 59.38% of the total, while males represented 40.63%. The age group of 31–35 was the most represented, accounting for 28.13%, followed by those aged 36–40 and 26–30. Regarding education level, 55.97% of respondents held a bachelor's degree or equivalent, followed by those with a college degree or equivalent. According to China's 2021 census data, the educational background distribution in Shandong is as follows: ~16% of the population has a university-level education (junior college or above), 16% have completed senior high school, 41% have attained a junior high school education, and 27% have received only primary school education (http://tjj.shandong.gov.cn/art/2021/5/21/art_156112_10287521.html). In contrast, in this study, 55.97% of the population had a bachelor's degree or equivalent, while an additional 13.64% held a master's degree or higher. The combined proportion of these two groups (69.61%) was substantially higher than the percentage of individuals with a college-level education in the general census population. A Pearson‘s chi-squared test was conducted to compare the educational distribution between the two groups. The results indicated that the proportion of individuals with higher education in the study population was significantly greater than in the general population (*X*^2^ = 745.13, *p* < 0.001), confirming a substantial overrepresentation of individuals with a bachelor's degree or higher.

Occupation-wise, the non-medical industry in enterprises and institutions was the most represented, accounting for 43.75%, followed by the medical industry in enterprises and institutions. As for types of medical insurance, employee medical insurance was the most common among respondents, accounting for 76.14%, followed by resident medical insurance. Concerning the place of permanent residence, the area where the local government is located had the most respondents, accounting for 52.84%. Regarding income level, the monthly income groups of 5,001–7,000 yuan and 7,001–9,000 yuan were most prominent, accounting for 24.43% and 19.89%, respectively (Table 1).

**Table 1:** Basic characteristics of the surveyed population.

**Item**	**Option**	**Frequency**	**Percentage (%)**	**Cumulative percentage (%)**
Gender	Male	143	40.63	40.63
	Female	209	59.38	100
Age	14–18	2	0.57	0.57
	19–25	27	7.67	8.24
	26–30	59	16.76	25
	31–35	99	28.13	53.13
	36–40	67	19.03	72.16
	41–45	35	9.94	82.1
	46–50	24	6.82	88.92
	51–55	15	4.26	93.18
	56–60	12	3.41	96.59
	61–70	9	2.56	99.15
	71+	3	0.85	100
Education level	Junior high school and below	16	4.55	4.55
	High school or equivalent	36	10.23	14.77
	College degree or equivalent	55	15.63	30.4
	Bachelor's degree or equivalent	197	55.97	86.36
	Master degree and above	48	13.64	100
Profession	Students-high school and below	4	1.14	1.14
	Students-college and above	12	3.41	4.55
	Enterprises and institutions in non-medical industries	154	43.75	48.3
	Enterprises and institutions medical industry	70	19.89	68.18
	Agricultural practitioners	6	1.7	69.89
	Self-employed persons	27	7.67	77.56
	Retirees	13	3.69	81.25
	Civil servant	15	4.26	85.51
	Unemployed, housewife	6	1.7	87.22
	Freelance	20	5.68	92.9
	Other	25	7.1	100
Medical insurance type	Resident medical insurance	66	18.75	18.75
	employee medical insurance	268	76.14	94.89
	business insurance	7	1.99	96.88
	No health insurance	5	1.42	98.3
	Other	6	1.7	100
Permanent residence	Rural area	17	4.83	4.83
	Township	33	9.38	14.2
	County and district government area	66	18.75	32.95
	The area where the local government is located	186	52.84	85.8
	Provincial capital	44	12.5	98.3
	Other	6	1.7	100
Monthly income	Student	16	4.55	4.55
	2,000 and below	15	4.26	8.81
	2,001–3,000	21	5.97	14.77
	3,001–5,000	66	18.75	33.52
	5,001–7,000	86	24.43	57.95
	7,001–9,000	70	19.89	77.84
	9,001–10,000	31	8.81	86.65
	10,001–15,000	39	11.08	97.73
	15,001–20,000	5	1.42	99.15
	20,000 and above	3	0.85	100
Number of children	0	71	20.17	20.17
	1	175	49.72	69.89
	2	94	26.7	96.59
	3 and more	12	3.41	100
Eldest child's age	0–3	26	7.39	27.56
	4–6	55	15.63	43.18
	7–12	101	28.69	71.88
	13–18	37	10.51	82.39
	19–25	25	7.1	89.49
	26–30	17	4.83	94.32
	31–35	8	2.27	96.59
	36–40	4	1.14	97.73
	41–45	6	1.7	99.43
	46–50	1	0.28	99.72
	51 and above	1	0.28	100
Age of the older parent	40 and below	1	0.28	0.28
	41–45	7	1.99	2.27
	46–50	11	3.13	5.4
	51–55	46	13.07	18.47
	56–60	80	22.73	41.19
	61–70	107	30.4	71.59
	71–80	57	16.19	87.78
	81 and above	27	7.67	95.45
	Other	16	4.55	100
Ability to use electronic devices (mobile phones, computers, etc.) to access the Internet	Yes	347	98.58	98.58
	No	5	1.42	100

### Analysis of “Internet + medical and health” platform usage

Upon comparing the usage of different levels and types of medical and health platforms by the respondents, it is evident that the hospital WeChat official account has the highest usage rate, accounting for 28.98%, followed by the hospital APP, which accounts for 21.02%. The usage rates of provincial and municipal platforms are relatively low, at 21.55% and 16.25%, respectively. The usage rates of third-party platforms are also relatively low, standing at only 12.01%; the usage rates of other types of platforms are the lowest, at a mere 0.18% (Table 2).

**Table 2:** Examination of the utilization of the “Internet + medical and health” platform by participants.

**Item (multiple choice)**	**Response**		**Penetration rate (*n* = 352)**
	***n***	**Response rate**	
Provincial platforms such as “Healthy Shandong Service Account”	122	21.55%	34.66%
Municipal platforms such as “Health Dezhou”	92	16.25%	26.14%
Hospital App	119	21.02%	33.81%
Hospital WeChat public account	164	28.98%	46.59%
Third-party platforms (such as Haodafu Online^a^, Xunyiwenyao.com^b^)	68	12.01%	19.32%
Others (please specify briefly)	1	0.18%	0.28%
Total	566	100%	160.80%

^a^https://www.haodf.com/; ^b^https://www.xywy.com/.

### Analysis of the reasons why respondents do not consider using “Internet + medical and health” services

There are various reasons as to why respondents do not consider Internet + healthcare services. Notably, over 13% of respondents reported challenges in operating Internet medical services, likely related to their level of digital literacy or technical confidence. Almost 18% of interviewees, steeped in the traditional model of diagnosis and treatment, expressed their dependence on and hesitation toward the traditional face-to-face approach. Meanwhile, almost 15% of respondents voiced their concerns over the privacy and security of their personal data, as online-based healthcare services can expose such data to breaches and unauthorized access. Consequently, they saw privacy protection as a paramount consideration for such adoption (Table 3).

**Table 3:** Analysis of the factors influencing respondents' reluctance to use “Internet + medical and health” services.

**Item (multiple choice)**	**Response**		**Penetration rate (*n* = 352)**
	***n***	**Response rate**	
Illiterate	13	2.00%	3.69%
Lack of electronic equipment	32	4.92%	9.09%
Lack of network environment	51	7.83%	14.49%
Difficult to operate	88	13.52%	25.00%
Don't know about the platform	102	15.67%	28.98%
Do not trust doctors' diagnosis and treatment skills	66	10.14%	18.75%
Accustomed to the traditional diagnosis and treatment model	117	17.97%	33.24%
The inability to effectively protect personal privacy	95	14.59%	26.99%
Laboratory test and examination cannot be carried out	72	11.06%	20.45%
Others (please specify briefly)	15	2.30%	4.26%
Total	651	100%	184.94%

### Service evaluation analysis

It scored more than four points on a scale of five, indicating that convenience in medical treatment is universally appreciated: 4.176; time-saving features over conventional practices should be appreciated: 4.193; and everyone kind of expected it to lower the cost of diagnosis and treatment: 3.733.

Nevertheless, the issue of trust still lingers around such systems. The survey results uncovered the apprehensions of the participants regarding patient information protection, thus scoring 3.557, closely followed by the protection of patient rights, with a score of 3.477. This implies that there should be further implementors to proceed and elaborate on some other security, even better regulation to warrant adherence to data privacy laws besides the ethical standards posed questionably in health care.

In terms of trends in development, there is a consensus among the respondents that Internet + health care services fit with the modern times of healthcare service provision, rated at 4.153. The respondents also opined that government policies, on the whole, support development to a large extent, as depicted by a mean score of 4.094. However, respondents stressed that in addition to framing the legal bonds surrounding digital health care, it's quite significant to reinstate clearer legal regulations articulating responsibilities upon all concerned stakeholders, scoring 4.330. Besides, respondents claimed that they often come to Tomato to promote Internet + health services with road-building a score of 3.764, and it was found that those in their social circles have started to grasp these services and undertake them: 3.832. At the same time, they expressed a strong desire for these services and willingness to maintain involvement with them, which earned a score of 4.094, while their wish to learn the needed skills to use them effectively scored 4.233. The results further suggest that more outreach for education, transparency in information on security processes, and precise regulatory measures would tackle lingering concerns confronting the widespread adoption of Internet + healthcare service (Table 4).

**Table 4:** The survey results on the recognition of “Internet + medical and health” services among the population.

**Items**	**Completely agree**	**Agree**	**Unclear**	**Disagree**	**Completely disagree**	**Score (Mean ±SD)**
“Internet + medical health” services make the medical treatment process more convenient	122	173	54	3	0	4.176 ± 0.710
“Internet + medical health” services can save more time than traditional medical methods.	126	174	46	6	0	4.193 ± 0.722
“Internet + medical health” services can reduce diagnosis and treatment costs	91	113	114	31	3	3.733 ± 0.971
“Internet + medical health” services can improve the medical environment	94	158	80	18	2	3.920 ± 0.864
“Internet + medical health” service platform and software are easily downloadable and user-friendly.	114	163	60	14	1	4.065 ± 0.823
I found it straightforward to learn and proficiently operate the “Internet + medical and health” services.	114	185	47	5	1	4.153 ± 0.720
Utilizing the “Internet + medical health” platform aids in assessing my medical condition.	80	154	107	6	5	3.847 ± 0.840
I harbor reservations regarding the accuracy of the diagnostic and therapeutic decisions made via the “Internet + medical health” service.	69	136	106	38	3	3.653 ± 0.942
It is challenging to safeguard patients' data while utilizing “Internet + medical and health” services.	59	125	127	35	6	3.557 ± 0.941
It is challenging to safeguard patients' rights while utilizing “Internet + medical and health” services.	64	109	122	45	12	3.477 ± 1.037
It is perceived that “Internet + medical health” services align with the contemporary trend of medical development.	114	185	47	5	1	4.153 ± 0.720
Government policies generally support the development of “Internet + medical and health” services.	117	165	60	6	4	4.094 ± 0.816
The implementation of “Internet + medical and health” services necessitates sound laws and regulations to ensure the responsibilities of all parties are clearly defined.	161	154	30	6	1	4.330 ± 0.728
The promotion of “Internet + medical and health” services is frequently observed.	83	149	76	42	2	3.764 ± 0.963
An increasing number of individuals in my vicinity have started to comprehend and utilize “Internet + medical and health” services.	87	155	81	22	7	3.832 ± 0.938
I am intrigued by “Internet + medical and health” services and intend to persistently monitor its progression.	103	190	50	7	2	4.094 ± 0.747
I aspire to acquire proficiency in utilizing “Internet + medical and health” services.	125	189	34	3	1	4.233 ± 0.677

SD, standard deviation.

The results showed no significant differences in Likert scale scores across gender, age, education, and income categories. However, a significant difference was found between individuals with a bachelor's degree or higher and those with a collegiate degree or lower (Table 5).

**Table 5:** The comparisons of Likert scale scores across gender, age, education, and income categories.

**Item**	**Option**	**Frequency**	**Median (Q1–Q3)**	***p* value**
Gender	Male	143	50 (45–53)	0.606
	Female	209	50 (45–56)	
Age	14–35	187	50 (46–57)	0.0717
	36+	165	49 (44–53)	
Education level	College degree and below	107	48 (43–52.5)	0.0466
	Bachelor's degree and above	245	50 (46–56)	
Income	5,000 and below	118	48.5 (43.25–55.75)	0.191
	5,001 and above	234	50 (46–54)	

## Discussion

Security surrounding personal data still remains one of the greatest barriers to total acceptance of Internet + healthcare services. Many users are unwilling to share their health data online owing to the risks involved with hacking, unauthorized third-party access, and an obscure way of handling data. The study showed that privacy-related concerns contributed significantly to the unwillingness of the users to engage with digital healthcare platforms, with 14.59% of its respondents flagging data security as the point of concern. Key reasons for not adopting digital healthcare platforms include the fear of data breaches, possible identity theft, and unauthorized persons' misuse of personal health information. The corresponding scientific literature backs these results, given that trust in data privacy, among other things, is a prerequisite for many patients to engage in digital health services (9, 11). Health platforms should, therefore, cater to those concerns so that user trust in such platforms can be bolstered through reforms, which include the use of end-to-end encryption, reliable user authentication methods, and transparent data management policies while adhering to established legal frameworks such as national healthcare information data protection regulations. Along with that, educational initiatives targeting users must also be prioritized so that they can learn about and appreciate the security measures in place, which will evoke more confidence in Internet + health services. Further research could incorporate qualitative interviews or focus groups concerning user perceptions and potential strategies to alleviate security concerns.

Internet usage often reflects an individual's level of concern for their health. A study examining the relationship between Internet use, healthcare service utilization, and self-rated health among older adults found a positive association between Internet use and self-rated health. Additionally, healthcare service utilization significantly influenced this relationship (23). For individuals with specific medical conditions, such as lupus erythematosus, recent surveys indicate that patients frequently use the Internet to seek health-related information, aiming to gain a comprehensive understanding of their disease and address unmet needs. This underscores the urgent need to develop high-quality digital resources to enhance patient education and promote disease self-management (24–27). The existing research highlights the national demand for Internet-based healthcare, the current gaps in its development, and the correlation between its advancement and patient health outcomes. While studies emphasize the importance of expanding Internet healthcare services, findings may vary based on users' medical backgrounds. For instance, research on medical students in China suggests that their top priorities for Internet-based healthcare services include “clinical services,” “decision support,” and “public health” (28). Conversely, individuals without a medical background tend to prefer non-professional medical websites, often relying on unregulated sources for health information. Overly technical medical websites may not be suitable for general users (29). This discrepancy further justifies the necessity of this study.

One of the key conclusions of this study is that security concerns are prevalent among populations engaging with Internet-based healthcare services. For instance, despite the U.S. federal government's efforts to promote the widespread adoption of electronic health records and the digitization of healthcare systems, data security remains a major concern for patients (30). These concerns are not merely intuitive but are supported by empirical evidence. A study examining health-related mobile applications (mHealth apps) found that 88.0% of such apps contain code capable of collecting user data, while 3.9% transmit user information through their traffic. This analysis revealed serious privacy risks and inconsistencies in privacy practices across mHealth apps (31). Privacy concerns are particularly significant in sensitive domains such as reproductive health applications, where users face heightened privacy risks (32).

Consequently, numerous research teams are actively working to enhance the security of Internet-based healthcare platforms. For example, Bao et al. (33) developed a verifiable and privacy-protected fine-grained data-sharing keyword search scheme, enabling efficient fine-grained access control, and ciphertext keyword search while improving information security. Similarly, Ali et al. (34) proposed a privacy-enhancing scheme that integrates homomorphic encryption with blockchain technology to strengthen privacy protection in IoT-based healthcare applications. These security measures primarily focus on protecting patient data—such as medical records, diagnostic information, and genetic data—from unauthorized access, misuse, or disclosure.

In addition to external threats, internal security concerns, such as insider violations and abuse of authority, also pose significant risks. Some healthcare platforms and apps actively collect patients' personal information, raising concerns about data exploitation. Establishing strong legal regulations is a necessary step toward mitigating these risks and restoring public trust. A recent study assessed compliance with privacy policies among Internet hospital applications in mainland China. The average compliance score for 52 evaluated apps was 73 out of 100, reflecting significant variations in adherence to privacy standards. Furthermore, only 12 applications required separate consent to process sensitive personal data. These findings highlight both the strengths and deficiencies of privacy policies in Internet hospital apps, underscoring the urgent need for stricter regulatory enforcement to safeguard users' personal information (35).

Certain strategies must be applied to ensure Internet + health services are efficiently implemented to overcome legal, cybersecurity, and user engagement challenges. Legal superstructure is another critical aspect of the attuning of faith in digital healthcare. A national framework for digital health security should be set up and tested against the standards set by HIPAA in the U.S., which regulates and requires certain security standards across platforms. Routine audits for security compliance should be enforced legally, while a designated governmental agency should supervise patient complaints and the application of legal standards for privacy (12, 36). Cybersecurity measures need to be enhanced tremendously to keep sensitive data away from cyber attackers. Some mechanisms to enhance user safety include full encryption for entire utilization, enforcing mandatory two-factor verification, and using great and strict penalties against violators of the data breach laws. In addition, launching interactive tutorials within hospital applications will advance the trust of patients with digital health services (8, 10). Internet + healthcare services' other critical part becomes incentivizing. That is to say, reimbursements in insurance for virtual consultations amuse the first-timer through reduced costs on telemedicine and make users aware of further engagement. Moreover, building strategic partnerships between health institutions, technology providers, and policymakers to devise common absorption frameworks responding to patient requirements and security concerns is a criterion for global acceptance of digital healthcare services (5, 37).

## Strengths and limitations

This study provides valuable empirical insights into the adoption and challenges of Internet + medical and health in Shandong, China. A major strength of this research is its large and diverse participant sample, allowing for a comprehensive quantitative analysis of user experiences and concerns. Additionally, the study employs rigorous statistical methods, such as reliability analysis (Cronbach's alpha = 0.912), ensuring the robustness of the findings.

However, some limitations must be acknowledged. First, this research was constrained by a tight schedule and demanding tasks. The number and scope of collected questionnaires and the depth of analysis could be improved. Second, self-reported data may introduce response bias, as participants might underreport or overreport concerns based on personal perceptions. Lastly, the study primarily focuses on patients from public medical institutions, potentially limiting generalizability to private healthcare users or rural populations.

## Future research directions

To build upon these findings, future research should consider longitudinal studies to analyze how perceptions of Internet+ healthcare evolve over time, particularly as new cybersecurity policies and legal frameworks emerge. Additionally, qualitative research—including in-depth interviews and focus groups—could provide richer insights into user concerns, motivations, and expectations beyond quantitative survey responses. Moreover, comparative studies across different provinces in China or international settings could offer a broader perspective on global best practices in digital healthcare adoption. Lastly, future studies should collaborate with technology developers and healthcare policymakers to evaluate the effectiveness of security measures and regulatory interventions in fostering public trust and engagement.

## Conclusion

This study successfully identified key barriers to the adoption of Internet Plus healthcare services, with data security concerns emerging as the primary deterrent. By validating the need for stronger legal oversight, advanced cybersecurity measures, and user education, the research resolves its core investigative issue.

## Data Availability

The raw data supporting the conclusions of this article will be made available by the authors, without undue reservation.
